# Supplementary material for: Iterative improvement in the automatic modular design of robot swarms
Source: PeerJ Comput Sci. 2020 Dec 7;6:e322. doi: 10.7717/peerj-cs.322 (PMC7924708; doi:10.7717/peerj-cs.322)
Supplement: Supplemental Information 3 [file peerj-cs-06-322-s003.zip › argos3/doc/api/standalone/a00596_source.html]

ARGoS: plugins/simulator/media/led\_medium.cpp Source File


- Main Page
- Related Pages
- Namespaces
- Classes
- Files

- File List
- File Members

# plugins/simulator/media/led\_medium.cpp

Go to the documentation of this file.

```
00001 #include "led_medium.h"
00002 #include <argos3/core/simulator/simulator.h>
00003 #include <argos3/core/simulator/space/space.h>
00004 #include <argos3/core/simulator/space/positional_indices/grid.h>
00005 #include <argos3/core/utility/configuration/argos_exception.h>
00006 #include <argos3/core/utility/logging/argos_log.h>
00007 
00008 namespace argos {
00009 
00010    /****************************************/
00011    /****************************************/
00012 
00013    CLEDMedium::CLEDMedium() {
00014    }
00015 
00016    /****************************************/
00017    /****************************************/
00018 
00019    CLEDMedium::~CLEDMedium() {
00020    }
00021 
00022    /****************************************/
00023    /****************************************/
00024 
00025    void CLEDMedium::Init(TConfigurationNode& t_tree) {
00026       try {
00027          CMedium::Init(t_tree);
00028          /* Get the positional index method */
00029          std::string strPosIndexMethod("grid");
00030          GetNodeAttributeOrDefault(t_tree, "index", strPosIndexMethod, strPosIndexMethod);
00031          /* Get the arena center and size */
00032          CVector3 cArenaCenter;
00033          CVector3 cArenaSize;
00034          TConfigurationNode& tArena = GetNode(CSimulator::GetInstance().GetConfigurationRoot(), "arena");
00035          GetNodeAttribute(tArena, "size", cArenaSize);
00036          GetNodeAttributeOrDefault(tArena, "center", cArenaCenter, cArenaCenter);
00037          /* Create the positional index for LED entities */
00038          if(strPosIndexMethod == "grid") {
00039             size_t punGridSize[3];
00040             if(!NodeAttributeExists(t_tree, "grid_size")) {
00041                punGridSize[0] = cArenaSize.GetX();
00042                punGridSize[1] = cArenaSize.GetY();
00043                punGridSize[2] = cArenaSize.GetZ();
00044             }
00045             else {
00046                std::string strPosGridSize;
00047                GetNodeAttribute(t_tree, "grid_size", strPosGridSize);
00048                ParseValues<size_t>(strPosGridSize, 3, punGridSize, ',');
00049             }
00050             CGrid<CLEDEntity>* pcGrid = new CGrid<CLEDEntity>(
00051                cArenaCenter - cArenaSize * 0.5f, cArenaCenter + cArenaSize * 0.5f,
00052                punGridSize[0], punGridSize[1], punGridSize[2]);
00053             m_pcLEDEntityGridUpdateOperation = new CLEDEntityGridUpdater(*pcGrid);
00054             pcGrid->SetUpdateEntityOperation(m_pcLEDEntityGridUpdateOperation);
00055             m_pcLEDEntityIndex = pcGrid;
00056          }
00057          else {
00058             THROW_ARGOSEXCEPTION("Unknown method \"" << strPosIndexMethod << "\" for the positional index.");
00059          }
00060       }
00061       catch(CARGoSException& ex) {
00062          THROW_ARGOSEXCEPTION_NESTED("Error in initialization of the LED medium", ex);
00063       }
00064    }
00065 
00066    /****************************************/
00067    /****************************************/
00068 
00069    void CLEDMedium::PostSpaceInit() {
00070       Update();
00071    }
00072 
00073    /****************************************/
00074    /****************************************/
00075 
00076    void CLEDMedium::Reset() {
00077       m_pcLEDEntityIndex->Reset();
00078    }
00079 
00080    /****************************************/
00081    /****************************************/
00082 
00083    void CLEDMedium::Destroy() {
00084       delete m_pcLEDEntityIndex;
00085       if(m_pcLEDEntityGridUpdateOperation != NULL) {
00086          delete m_pcLEDEntityGridUpdateOperation;
00087       }
00088    }
00089 
00090    /****************************************/
00091    /****************************************/
00092 
00093    void CLEDMedium::Update() {
00094       m_pcLEDEntityIndex->Update();
00095    }
00096 
00097    /****************************************/
00098    /****************************************/
00099 
00100    void CLEDMedium::AddEntity(CLEDEntity& c_entity) {
00101       m_pcLEDEntityIndex->AddEntity(c_entity);
00102    }
00103 
00104    /****************************************/
00105    /****************************************/
00106 
00107    void CLEDMedium::RemoveEntity(CLEDEntity& c_entity) {
00108       m_pcLEDEntityIndex->RemoveEntity(c_entity);
00109    }
00110 
00111    /****************************************/
00112    /****************************************/
00113 
00114    REGISTER_MEDIUM(CLEDMedium,
00115                    "led",
00116                    "Carlo Pinciroli [ilpincy@gmail.com]",
00117                    "1.0",
00118                    "Manages the LEDs.",
00119                    "This medium is required to manage the LED entities, thus allowing the\n"
00120                    "associated camera sensors to work properly. If you intend to use a camera\n"
00121                    "sensor that detects colored blobs, you must add this medium to the XML\n"
00122                    "configuration file.\n\n"
00123                    "REQUIRED XML CONFIGURATION\n\n"
00124                    "<led id=\"led\" />\n\n"
00125                    "OPTIONAL XML CONFIGURATION\n\n"
00126                    "None for the time being\n",
00127                    "Under development"
00128       );
00129 
00130    /****************************************/
00131    /****************************************/
00132 
00133 }
```

---

Generated on 10 Jul 2018 for ARGoS by 
 1.6.1
